# Potential biomarkers for early detection of pancreatic ductal adenocarcinoma

**DOI:** 10.1007/s12094-020-02372-0

**Published:** 2020-05-23

**Authors:** D. Kriz, D. Ansari, R. Andersson

**Affiliations:** Department of Surgery, Clinical Sciences Lund, Skåne University Hospital, Lund University, Lund, Sweden

**Keywords:** Pancreatic cancer, Early detection, Biomarkers, Sensitivity, Specificity

## Abstract

Pancreatic cancer has the highest mortality amongst all major organ cancers. Early detection is key to reduce deaths related to pancreatic cancer. However, early detection has been challenged by the lack of non-invasive biomarkers with enough sensitivity and specificity to allow for screening. The gold standard is still carbohydrate antigen (CA 19-9), against which all new biomarkers must be evaluated. In this paper, we describe recent progress in the development of new pancreatic cancer biomarkers, focusing on proteins, metabolites, and genetic and epigenetic biomarkers. Although several promising biomarkers have been identified, they are all derived from retrospective studies and additional prospective studies are needed to confirm their clinical validity.

## Introduction

Pancreatic ductal adenocarcinoma (PDAC), commonly known as pancreatic cancer, has the highest mortality rate of all major cancers. Despite many years of experimental research and clinical trials, the 5-year survival rate for pancreatic cancer is still less than 5% when all stages are considered [1]. The major reason for the poor survival is due to late detection. By the time the cancer is detected, it is usually locally advanced or metastatic. It is still unknown whether pancreatic cancer is a stepwise process with metastasis occurring late [2] or whether metastasis occurs early in neoplastic transformation [3]. Nevertheless, most agree that detecting pancreatic cancer in resectable stages is still the first step in any early detection strategy [4].

Carbohydrate antigen (CA) 19-9 is the only clinically used serum biomarker for pancreatic cancer. It is elevated in approximately 80% of all pancreatic cancer patients [5]. However, CA 19-9 has a low specificity and sensitivity in asymptomatic patients, and thus can merely be used for disease monitoring rather than early detection. Therefore, new biomarkers are needed.

A multitude of biomarkers have been proposed for early detection of pancreatic cancer derived from blood, tissue, pancreatic juice, saliva and urine. They can be classified into proteins, metabolites, and genetic and epigenetic biomarkers, including panels thereof. In this review, we provide a comprehensive summary of new non-invasive biomarkers for pancreatic cancer published during the last 5 years and their performance and clinical utility concerning early detection.

## Blood-based biomarkers

Blood is likely the most accessible biofluid for non-invasive, early detection. Many promising blood-based biomarkers have been identified for early-stage pancreatic cancer (Tables 1, 2, 3, 4).

**Table 1:** Protein biomarkers in blood

Biomarkers	Sensitivity (%)	Specificity (%)
CA 19-9 (up to 1 year prior to diagnosis) [7]	68	95
CA 19-9 (between 1 and 2 years prior to diagnosis) [7]	53	95
CA 19-9 + CA125 (up to 1 year prior to diagnosis) [7]	57.1	90
MIC-1 [8]	94	45.8
ADH [8]	62	83.3
Osteonectin [9]	84.6	87.5
IGFBP2 [10]	68.4	67.7
IGFBP3 [10]	76.3	70.7
Glypican-1 positive exosomes [12]	100	100
Apo-AII ATQ/AT + CA 19-9 [11]	95.4	98.3
TFPI + TNC + CA 19-9 [13]	76	84
TFF1 + TFF2 + TFF3 [14]	73	54
TFF1 + TFF2 + TFF3 + CA 19-9 [14]	85	92
Antibody microarray (29 markers) [15]	95	94
Serum electrospray mass profiling [16]	95	96
Protein corona-based test [17]	85	100
Autoantibodies against TAA [18]	50	90

**Table 2:** Metabolite biomarkers in blood

Biomarkers	Sensitivity (%)	Specificity (%)
Model X (1,5-AG, histidine, inositol, and xylitol) [19]	74.1	86
Model Y (histidine and xylitol) [19]	70.4	89.5
Model Y + CA 19-9 [19]	90.7	89.5
5-Hydroxytryptophan + LysoPE(18:2) + PC(16:0/16:0) + PC(18:0/22:4) + PE(17:0/0:0) + SM(d18:1/16:0) [20]	90	85

**Table 3:** Genetic and epigenetic biomarkers in blood

Biomarkers	Sensitivity (%)	Specificity (%)
KRAS exoDNA [21]	75.4	92.6
HYAL2A methylation [22]	75.6	93.7
2 miRNA panel [23]	79	85
PaCIC + miRNA [24]	100	80
7-lncRNA signature [25]	84	57.1

**Table 4:** Proteogenomic biomarkers in blood

Biomarkers	Sensitivity (%)	Specificity (%)
CancerSEEK [26]	72	99

### CA 19-9

The CA 19-9 assay measures a carbohydrate antigenic determinant that is expressed on various carrier proteins, including mucin proteins MUC1, MUC5AC, and MUC16 [6]. CA 19-9 is reported to have a sensitivity of 68% and specificity of 95% one year prior to diagnosis, and a sensitivity of 53% two years prior to diagnosis of PDAC [7]. The combination of CA 19-9 and CA125 provides a sensitivity of 57.1% with a specificity of > 90% up to one year before the diagnosis of PDAC.

### Protein biomarkers

#### Individual protein markers

Circulating macrophage inhibitory cytokine (MIC-1) and alcohol dehydrogenase (ADH) have been evaluated for detection of early-stage PDAC. MIC-1 had a 94% sensitivity at 45.8% specificity, while ADH had a sensitivity of 62% at 83.3% specificity [8]. Combining MIC-1, ADH and CA 19-9 significantly improved the area under the curve (AUC) to 0.89 for detection of early-stage pancreatic cancer.

Osteonectin, also known as secreted protein acidic and rich in cysteine (SPARC), is a secreted, phosphorylated, calcium-binding glycoprotein. Osteonectin has a sensitivity of 84.6% and a specificity of 87.5% for detection of early-stage pancreatic cancer [9].

Insulin like growth factor binding protein 2 (IGFBP2) and IGFBP3 are also potential biomarkers for early-stage pancreatic cancer. IGFBP2 was reported to have a sensitivity of 68.4% with a specificity of 67.7%, while IGFBP3 was reported to have a sensitivity of 76.3% and a specificity of 70.7% [10]. Combining these biomarkers with CA 19-9 resulted in an increased effectiveness of detection, with an AUC of 0.90.

Levels of the apolipoprotein AII isoform Apo-AII ATQ/AT were found to be decreased in patients with pancreatic cancer. The sensitivity of Apo-AII-ATQ/AT combined with CA 19-9 was 95.4% with a specificity of 98.3% [11].

However, most impressively is the study of glypican-1 on cancer-cell-derived exosomes, which was found to diagnose early-stage pancreatic cancer with absolute precision [12].

#### Protein panels and signatures

A panel consisting of tissue factor pathway inhibitor (TFPI), tenascin C (TNC-FN III-C) and CA 19-9, was found to improve the diagnostic performance compared to CA 19-9 alone. The sensitivity for this combination of biomarkers was 76% at a specificity of 84% [13].

Trefoil factors (TFF) are small, secretory mucin-associated proteins. The combination of TFF1, TFF2, and TFF3 yielded a sensitivity of 73% and a specificity of 54% for detection of early-stage pancreatic cancer [14]. Furthermore, combining the three trefoil factors with CA 19-9 resulted in a sensitivity and specificity of 85% and 92%, respectively.

An antibody microarray platform was developed to identify a serum biomarker signature for early-stage PDAC. This signature, consisting of 29 biomarkers, was found to detect stage I-II pancreatic cancer with an AUC of 0.96 with a sensitivity of 95% at 94% specificity [15].

The profiling of pancreatic cancer sera can also be performed using electrospray ionization mass spectrometry. The method provided a sensitivity of 95% at a specificity of 96% [16]. For later stages of PDAC the test had a sensitivity and specificity of 100%.

A novel protein-corona blood test was recently presented in order to detect PDAC in its early stages. The method utilizes nano-biointeractions between nanoparticles and blood samples. The results showed that the blood test had a sensitivity of 85% and specificity of 100% [17].

#### Circulating autoantibodies

Circulating autoantibodies against tumor associated antigens (TAA) have been used as diagnostic biomarkers for pancreatic cancer. Most autoantibodies (85%) showed a low sensitivity (< 50%), but a high specificity (> 90%) [18].

### Metabolite biomarkers

Histidine, xylitol, 1,5-anhydro-d-glucitol (1,5-AG), and inositol have been investigated as potential biomarkers for early-stage pancreatic cancer [19]. The metabolites were divided into model X and model Y, where Model Y included histidine and xylitol and model X included all four mentioned metabolites. The results showed that Model X had a sensitivity of 74.1% and a specificity of 86%, Model Y had a sensitivity and specificity of 70.4% and 89.5%, respectively, and combining model Y with CA 19-9 resulted in a sensitivity of 90.7% and a specificity of 89.5%.

Another study evaluated a six metabolite biomarker panel consisting of 5-hydroxytryptophan, LysoPE(18:2), PC(16:0/16:0), PC(18:0/22:4), PE(17:0/0:0), and SM(d18:1/16:0) [20] for early detection of PDAC. The six metabolite panel had a sensitivity of 90% and a specificity of 85%.

### Genetic and epigenetic biomarkers

PDAC is hallmarked by commonly mutated genes such as KRAS. The diagnostic performance of mutant KRAS in circulating exosome DNA from patients with early stage PDAC was reported to be 75.4% with a specificity of 92.6% [21].

PDAC also harbors epigenetic modifications including changes in DNA methylation, histone components, and non-coding RNAs, specifically microRNA (miRNA) expression and long noncoding RNAs (lncRNAs).

The diagnostic potential of DNA methylation in the hyaluronoglucosaminidase 2 (HYAL2) gene was recently evaluated [22]. The sensitivity and specificity for diagnosing pancreatic cancer were 75.6% and 93.7%, respectively.

miRNA biomarkers have been extensively evaluated as early diagnostic biomarkers in pancreatic cancer. A meta-analysis found that 32 miRNAs were upregulated in PDAC patients, while 5 miRNAs were downregulated [23]. The highest diagnostic performance was achieved using 2 miRNA panels with a sensitivity of 79% and specificity of 85%.

Serum-exosome miRNA and protein markers have been studied for the early detection of PDAC. Four selected miR markers (miR-1246, miR-4644, miR-3976, and miR-4306) provided a sensitivity of 96% at 86% specificity. The sensitivity and specificity of pancreatic cancer-initiating cell markers (PaCIC) CD44v6, Tspan8, EpCAM, MET and CD104 were 81% and 94%, respectively [24]. The combination of miRNA and PaCIC markers resulted in a sensitivity and specificity of 100% and 80%, respectively.

LncRNA biomarkers for early detection of PDAC have also been examined, including a 7-lncRNA signature [25]. The sensitivity and specificity of the 7-lncRNA signature were 72.2% and 33.3%, respectively.

### Proteogenomic biomarkers

CancerSEEK [26] utilizes genetic and protein biomarkers in order to detect surgically resectable cancers as well as localize organs of origin. The sensitivity ranged from 69 to 98% at 99% specificity for the different tumors, including pancreatic, ovary, liver, stomach, esophagus, colorectal, lung and breast cancers. For pancreatic cancer, the sensitivity of the detection method was 72% at 99% specificity.

## Salivary biomarkers

Saliva is another potential source of non-invasive biomarkers, with many candidates having been identified (Table 5).

**Table 5:** Salivary biomarkers

Biomarkers	Sensitivity (%)	Specificity (%)
KRAS + MBD3L2 + ACRV1 + CDKL3 [27]	71	69
29 biomarkers [27]	87	85
HOTAIR + PVT1 [28]	78.2	90.9

Four salivary mRNA biomarkers (KRAS, MBD3L2, ACRV1 and CDKL3) were found to have a sensitivity of 71% with a specificity of 69% [27]. In the study, 29 additional biomarkers (ACRV1, AGO1, BUB1, CA2, COL1A2, ESR1, EVL, FOXC1, GLUL, H2AFX, HES1, HIST1H2BD, KRAS, MAPK6, MBD3L2, MCM7, MGMT, NCOA2, NR5A1, PDGFRA, RNASEH2A, RPL22, SOD2, SRSF1, STAT5B, SULT2A1, TAF8, VEGFA, and WDFY2) were discovered that all together had a sensitivity of 87% and a specificity of 85%.

Another study investigated the possibility of using salivary HOX transcript antisense intergenic RNA (HOTAIR) and plasmacytoma variant translocation 1 (PVT1) as potential biomarkers for early detection of PDAC [28]. The HOTAIR and PVT1 sensitivity was 78.2% with a specificity of 90.9%.

## Urine biomarkers

Urine can also be used to detect non-invasive pancreatic cancer biomarkers (Table 6).

**Table 6:** Urine biomarkers

Biomarkers	Sensitivity (%)	Specificity (%)
LYVE-1 + REG1A + TFF1 [29]	76.9	89.8
miR-143 [30]	83.3	88.5
miR-30e [30]	83.3	80.8
miR-223 [30]	83.3	76.9
miR-143 + miR-30e [30]	83.3	96.2
miR-30e + miR-223 [30]	83.3	92.3

A three marker panel (LYVE-1, REG1A and TFF1) in urine was recently evaluated [29]. The sensitivity and specificity of the three biomarkers combined were 80% and 76.9%, respectively.

Three urinary miRNA biomarkers (miR 143, miR-30e, and miR-223) were evaluated in another study [30]. The sensitivity of the combination of miR-143+miR-30e was 83.3% and the specificity 96.2%, while the combination of miR-30e+miR-223 had a 83.3% sensitivity at 92.3% specificity.

## Conclusion

Early detection is probably the most important strategy to reduce mortality rates in pancreatic cancer. However, the lack of biomarkers with clinical utility remain a major problem. Other than CA 19-9, no marker is used in the routine clinical management of pancreatic cancer. Multiple investigational biomarkers for pancreatic cancer have been discovered in recent years, with promising diagnostic performance. However, it seems evident that single markers generally do not have enough accuracy to detect early-stage pancreatic cancers and panels or even biomarker signatures may become necessary. As all the biomarkers presented herein were developed from retrospective studies, additional validation studies in larger cohorts are needed to corroborate these finding. In these validation studies it will be important to use pre-diagnostic samples and samples from high-risk patients in order to prove clinical utility.
